# Opinion dynamics in social networks under competition: the role of influencing factors in consensus-reaching

**DOI:** 10.1098/rsos.211732

**Published:** 2022-05-18

**Authors:** Ningning Lang, Lin Wang, Quanbo Zha

**Affiliations:** School of Management Science and Real Estate, Chongqing University, Chongqing 400044, People's Republic of China

**Keywords:** opinion dynamics, competition, consensus, social network

## Abstract

The rapid development of information technology and social media has provided easy access to the vast data on individual preferences and social interactions. Despite a series of problems, such as privacy disclosure and data sensitivity, it cannot be denied that this access also provides beneficial opportunities and convenience for campaigns involving opinion control (e.g. marketing campaigns and political election). The profitability of opinion and the finiteness of individual attention have already spawned extensive competition for individual preferences on social networks. It is necessary to investigate opinion dynamics over social networks in a competitive environment. To this end, this paper develops a novel social network DeGroot model based on competition game (DGCG) to characterize opinion evolution in a competitive opinion dynamics. Social interactions based on trust relationships are captured in the DGCG model. From the model, we then obtain equilibrium results in a stable state of opinion evolution. We also analyse what role relevant factors play in the final consensus and competitive outcomes, including the resource ratio of both contestants, initial opinions, self-confidence and network structure. Theoretical analyses and numerical simulations show that these factors can significantly sway the consensus and even reverse competition outcomes.

## 1. Introduction

Recent advances in information technology have provided easy access to information regarding the preferences of individuals and enabled structural data on their interaction to be recorded [1–3]. Furthermore, increasingly comprehensive privacy policies promote people's willingness to share information [3]. Individuals usually share and communicate their opinions, thoughts and preferences over social networks. The choice behaviours of people are unprecedentedly influenced by the preference information of others in real decision-making problems. This phenomenon is widely studied in opinion dynamics, which refer to the evolution process of opinions among a group of agents through social interaction.

Opinion dynamics are manifest in diverse application areas [4,5], including marketing campaigns [1,6], public sentiment [7,8], political elections [9,10] and group decision-making [11,12]. These studies can be summarized under five topics: (i) opinion dynamics in different expression formats [13–15], (ii) modelling different evolution rules [16–18], (iii) opinion dynamics in heterogeneous environments [19–21], (iv) strategies and optimization for opinion control [22–24], and (v) opinion dynamics integrated with traditional group decision-making problems [25–27]. Some researchers are also devoted to empirical and applied studies of the above topics [28–30], which in turn enriches the theory of opinion dynamics.

Opinions reflect people's preferences and behavioural tendencies. The collection and use of such data can benefit lobbyists or marketers in enabling a quick response to the election or market [3]. Although this process has aroused public concerns over information privacy and security, it has not been concluded that this privacy concern will definitely inhibit people from sharing personal information [2,31]. As a result, the competition for opinions is usually profitable and widespread in social networks [32,33]. Naturally, social network opinion dynamics in monopolistic or competitive environments has attracted the attention of researchers in recent years. Tavasoli *et al*. [30] explored the incentive rate determination during a viral marketing campaign and investigated the dynamics of agents' discrete states over a social network in a monopolistic environment. Luo *et al.* [34] studied the dynamics of opinions through numerical simulations under the effect of monopolistic advertising. Varma *et al*. [15] studied the consensus-reaching process over social networks in the presence of an external monopolist. Morarescu *et al.* [35] addressed how to sway the collective opinion of agents in a social network toward the desired opinion by using the fixed budget of a monopolistic marketer. Moreover, an influence-maximization problem under a competitive environment was studied in Kahr *et al.* [36]. Guzmán [37] investigated the opinion dynamics in a competition for individual support between two parties. Bimpikis *et al*. [1] studied the problem of competitive targeted advertising for new products over a social network. Among them, the work of Guzmán [37] is closely related to ours. Despite similarly applying the DeGroot model and contest success functions (CSFs), the social network in his paper was treated as a black-box. Guzmán did not engage in deeper discussion on how people interact to update their opinions in the network and what factors affect the process. Therefore, although several achievements have been made, there are still some limitations to be resolved in the research on consensus-reaching in opinion dynamics under competitive environments:

(i) Although the consensus in stable state has been widely explored in opinion dynamics [4,15,23,26,35,38], its results for a competitive environment are rarely investigated. Because intense competition for individual attention and preferences in social networks exists, it is necessary to study opinion dynamics over social networks in a competitive environment.
(ii) Some equilibrium results have been presented in studies on opinion dynamics under competition [1,37,39]; however, the results do not reveal clearly how the relevant factors affect the consensus in such competitive opinion dynamics.

Motivated by the above limitations, this paper aims to investigate opinion evolution and consensus-reaching over social networks in a competitive environment, and analyse what role relevant factors play in the consensus and competitive outcomes. It should be noted that the use of personal data can be a double-edged sword. This paper highlights its advantages in order to provide insights into opinion competition strategies for decision-makers. However, its disadvantages, such as privacy disclosure and data sensitivity, are not our focus. The main contributions of this paper are as follows:

(1) From the perspective of psychology, we captured the self-confidence (trust in oneself) of individuals and modelled the structure of their social interactions based on the trust relationship. In doing so, we developed a social network DeGroot model based on competition game (DGCG) to characterize consensus-reaching in opinion dynamics under competition.
(2) We derived the analytic expression of stable equilibrium results from the DGCG model, including the final consensus in the social network and the resources allocated to each agent from contestants in the stable state. Our model explored the black-box of social interactions, and its results clearly reveal several influencing factors including network structure.
(3) Through theoretical analyses and numerical simulations on the equilibria, we found that the factors of contestants’ resource ratio, agents' self-confidence and initial opinions as well as network structure can significantly sway the consensus and even reverse competition outcomes.

The remainder of this paper is organized as follows: §2 provides an introduction to some preliminaries regarding the DeGroot model and the CSF; §3 describes the DGCG model and presents its stable equilibrium results; the role of the resource ratio, initial opinions and network structure in the consensus as well as competition outcomes is analysed in §4; finally, the discussion and conclusion are presented in §5.

## 2. Preliminaries

This section presents preliminary knowledge about the DeGroot model and the CSF, which is set as a cornerstone of the later sections.

### 2.1. DeGroot model

In continuous opinion dynamics, the DeGroot model is regarded as a classical model in general. DeGroot [40] specifies the conditions to reach a consensus and determines the consensus weight of each agent.

A given group of agents, *V* = {*v*_1_, *v*_2_, …, *v_n_*} comprises independent individuals who have no social relationship with each other. Let *t^i^* ∈ (0, 1) be the opinion of agents *v_i_* at time *t*, and let e*_ij_* ∈ (0, 1) be the trust weight that agent *v_i_* assigns to agent *v_j_*. It is assumed that *e_ij_* does not change over time or with opinions and $\sum_{\;j=1}^{n}e_{ij}=1$ for each agent *v_i_*. Then, the opinion evolution of agent *v_i_* can be described by

2.1 $$p_{i}^{t+1}=e_{i1}p_{1}^{t}+e_{i2}p_{2}^{t}+\cdots +e_{in}p_{n}^{t},\;t=0,1,2\ldots$$

Furthermore, the opinion evolution of the group can be written as

2.2 $$P^{\left(t+1\right)}=E\times P^{\left(t\right)}=E^{t}\times P^{\left(1\right)},\;t=0,1,2\ldots$$

where *E* = (e*_ij_*)*_n_*_×_*_n_* and $P^{\left(t\right)}={\left(\;p_{1}^{t},\;p_{2}^{t},\;\ldots ,\;p_{n}^{t}\right)}^{T}\in {\left(0,\;1\right)}^{n}$.

All opinions of agents will fuse into a consensus *C*, i.e. $\underset{t\to \infty }{\lim}p_{i}^{t}=C$ for *i* = 1, 2, … , *n*, if and only if the matrix power E^t^*(t* ∈ {0, 1, 2,…}) contains at least one strictly positive column [38]. Moreover, when a consensus is reached in the DeGroot model, the consensus *C* can be determined by equation (2.3).

2.3 $$C=\overset{n}{\underset{i=1}{\sum }}w_{i}p_{i}^{0},$$

where $\underset{t\to \infty }{\lim}E_{i}^{t}=W$ for *i* = 1, 2,… , *n*, i.e. *WE* = *W* and *E_i_* is the *i*-th row of the matrix *E^t^*, and *W* = (*w*_1_, *w*_2_, … , *w_n_*), $\overset{n}{\underset{i=1}{\sum }}w_{i}=1$.

We define *w_i_* ∈ (0, 1) as the consensus weight in this paper, which implies that the consensus *C* is a linear combination of the agents’ initial opinions.

Social network DeGroot (SNDG) model is a continuation of the DeGroot model. The SNDG model incorporates social relationship into the DeGroot model, so as to explore the consensus-reaching process of agents in a social network. Dong *et al*. [23] first proposed the SNDG model and emphasized the role of leadership in opinion dynamics over social networks. Based on this, Ding *et al*. [38] determined the consensus weights of opinion leaders in the reached consensus and derived the analytic expression of consensus weight in an undirected graph.

### 2.2. Contest success function

CSFs are applied to characterize a contest, wherein contestants compete for a prize by making irreversible efforts. Increased effort of each contestant improves his/her probability of winning, while reducing the opponents' chances [41]. The CSF is widely used in fields of political election [37,42,43], sports events [44–46] and marketing management [1,6,47], and many different variants of it have been developed over the long-term course of its application [48,49]. To the best of our knowledge, the CSF was first proposed by Luce to explore individual choice behaviour [50]. To date, there are two classic variants of the CSF dominating the related literature, namely the auction CSF and the lottery CSF [51,52]. The auction CSF assumes that the greatest effort is certain to win the prize, while the lottery CSF assumes the chance of success is proportional to effort: this is the essential distinction between the two variants. For the case of two contestants labelled *A* and *B*, *r_i_*(*i* ∈ {*A*, *B*}) denotes the efforts of contestant *i*, and *p_i_*(*r_A_*, *r_B_*) denotes the winning probability of contestant *i* (*i* ∈ {*A*, *B*}). If the opinion is continuous and closely bound up with the winning probability, the lottery CSF can be applied to map the effort level into the opinion space in contests [1,37,43,51]. Thus, *p_i_*(*r_A_*, *r_B_*) can be regarded as the opinion or preference for contestant *i* (*i* ∈ {*A*, *B*}). Based on the social exchange theory [53], the opinion of contestants can be improved by their efforts, which is manifested as a social exchange between them. Meanwhile, in order to accommodate asymmetric effects of contestant efforts on the opinions, the lottery CSF usually takes the following form.

2.4 $$p_{i}(r_{A},r_{B})=\frac{\alpha_{i}{\left(r_{i}\right)}^{\gamma }}{\alpha_{A}{\left(r_{A}\right)}^{\gamma }+\alpha_{B}{\left(r_{B}\right)}^{\gamma }},$$

where *α_i_* > 0 for ∀ i∈{A,B}, with *α_A_* > *α_B_* indicating that contestant *A* has an advantage over contestant *B* in turning his effort into opinion or preference. *γ* is the efficiency parameter of effort. 0 < *γ* ≤ 1 is generally used to ensure the existence of pure-strategy Nash equilibrium [1,54].

## 3. Social network DeGroot model based on competition game

**Figure 1.**
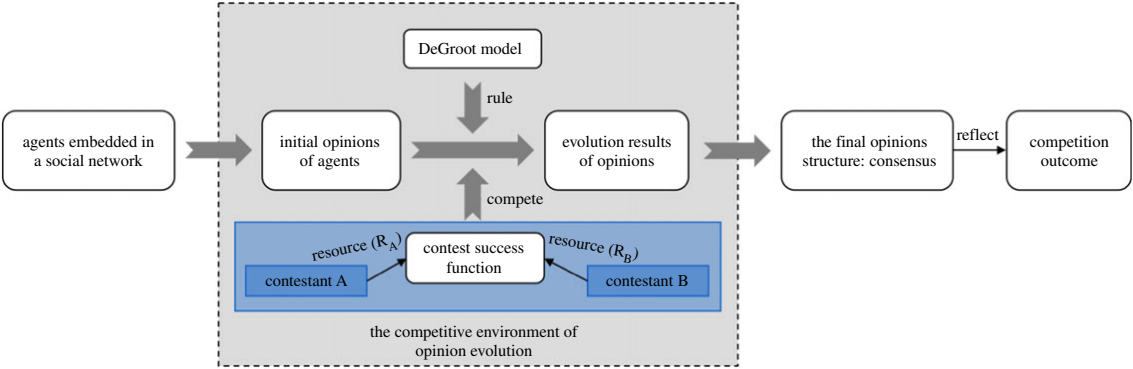
The framework of the DGCG model.

In view of the widespread competition phenomenon in opinion dynamics, a social network DGCG is developed in this section to investigate opinion evolution over social networks in a competitive environment. First, this section creates a new SNDG that accommodates heterogeneous trust between friends in order to appropriately model the evolution of opinion with social interactions. Second, a lottery CSF is incorporated into the DGCG model to investigate opinion evolution in the context of competition. During the process of opinion evolution, the opinion of each agent is influenced not only by the external interaction with his friends, but also by the competitive expenditure of external contestants simultaneously. For tractability and simplification of the exposition, we use two parts to introduce opinion evolution and competitive expenditure in the DGCG model as shown in figure 1.

### 3.1. Opinion evolution over the social network

In this paper, a social network is indicated by an undirected graph *G*(*V*, *E*) consisting of a set *V* of nodes and a set *E* of edges. *V* = {*v*_1_, *v*_2_, … , *v_n_*} is the set of agents embedded in the social network, and the set of *E* denotes social relations between these agents. Likewise, the adjacency matrix *A* = (*a_ij_*)*_n_*_×_*_n_* can also represent the connection relationship between agents. If (*v_i_*, *v_j_*) ∈ *E*, the value of *a_ij_* is taken as 1, otherwise it is 0. Assume that *G*(*V*, *E*) is a connected undirected graph without loops and parallel edges. The degree *d_i_* of an agent *v_i_* in *G*(*V*, *E*) refers to the number of friends that agent *v_i_* has. Each agent *v_i_* is endowed with an opinion $p_{i}^{t}\in (0,1)$ at time *t*(*t* ∈ {0, 1, 2, … , ∞}), which will be influenced by the opinions of his/her friends during interactions.

Opinion evolution is a process of fusion of agents’ opinions, in which agents interact with their friends and continuously update their opinions to reach a consensus, polarization (two conflicting or different opinions) or fragmentation (several clusters of different opinions) in the final stage [4,5,55]. Suppose that during the opinion evolution process, each agent is not only influenced by his/her friends in the network, but also maintains his/her own opinions to some extent. Let *β_i_* ∈ (0, 1) characterize the self-confidence of agent *v_i_* [38]. Then, (1 − *β_i_*) refers to the sum of trust in his/her friends, i.e. the sum of friends' influence. Note that the degree *d_i_* is used to reflect the influence of agent *v_i_* in *G*(*V*, *E*), namely degree centrality. The larger the degree of a node, the more influential it is in the network [30,56]. Furthermore, an agent with great influence deserves more trust [57]. For a given agent *v_i_*, he/she places different trust weights among his/her friends according to their degree centrality, which is an extension of Ding *et al*. [38]. Thus, the trust weight that agent *v_i_* places on his friend *v_j_* is

3.1 $$\omega_{ij}=\frac{(1-\beta_{i})a_{ij}d_{j}}{\sum_{\;j=1,j\neq i}^{n}a_{ij}d_{j}},\;j\neq i,i\in V.$$

Then, opinion evolution is carried out on the social network as follows:

3.2 $$p_{i}^{t+1}=\beta_{i}p_{i}^{t}+\overset{n}{\underset{\;j=1,j\neq i}{\sum }}\omega_{ij}p_{j}^{t}.$$

Equation (3.2) can be equivalently written as

3.3 $$P^{\left(t+1\right)}=M\times P^{\left(t\right)}=M^{t}\times P^{\left(1\right)},\;t=0,1,2\ldots ,$$

where $P^{\left(t\right)}={\left(\;p_{1}^{t},\;p_{2}^{t},\;\ldots ,\;p_{n}^{t}\right)}^{T}\in {\left(0,\;1\right)}^{n}$ and $M=\left[\begin{array}{c} \begin{array}{cc} \beta_{1} & \omega_{12}\\ \omega_{21} & \beta_{2}\\ \vdots & \vdots \end{array}\;\;\;\;\;\begin{array}{cc} \cdots & \omega_{1n}\\ \cdots & \omega_{2n}\\ \ddots & \vdots \end{array}\\ \begin{array}{cc} \omega_{n1} & \omega_{n2} \end{array}\;\;\;\;\;\begin{array}{cc} \cdots & \beta_{n} \end{array} \end{array}\right]$.

**Lemma 3.1**

[23]. *In regard to opinion evolution over the social network G(V, E), all agents will reach a consensus if and only if there exists a v_j_ ∈ V, and there is a directed path in the social network from v_i_ to v_j_ for all v_i_ ∈ V/{v_j_}.*

In this paper, we study opinion evolution over an undirected social network *G*(*V*, *E*), where any agent can be reached from any other agent via an undirected path. Therefore, agents in *G*(*V*, *E*) are sure to reach a consensus in the final stage of equation (3.2), and the weight of each agent in the consensus is shown as proposition (3.1).

**Proposition 3.1.** *Given an undirected graph/social network G(V, E), agents interact with each other as per equation (3.2); then, a consensus is reached in accordance with equation (2.3), with the consensus weight of agent v_i_ being*

3.4 $$w_{i}=\frac{\left(\sum_{\;j=1,j\neq i}^{n}a_{ij}d_{j}\right)d_{i})/1-\beta_{i}}{\sum_{i=1}^{n}\left\{\left(\sum_{\;j=1,j\neq i}^{n}a_{ij}d_{j}\right)d_{i}/1-\beta_{i}\right\}},$$

*where w_i_ is non-negative and* $\sum_{i=1}^{n}w_{i}=1$*.*

*Proof.* The proof of proposition (3.1) is provided in the appendix A.

Proposition (3.1) reveals that the agent with many friends, who in turn also have many friends, generally holds a large share in the consensus *C*. Meanwhile, the more self-confident the agent is, the larger weight he/she has in the consensus.

### 3.2. Shaping initial opinions by competitive expenditure

There are two contestants, *A* and *B*, with costless resources, *R_A_* and *R_B_*, that can be spent upon agents to shape their opinions. The opinions can be interpreted as support in campaign [37], brand awareness in marketing [1] and market share in business [32]. In these cases, the benefits of both contestants are closely related to the collective opinion of all agents [5]. Suppose that each contestant is rational economic man, and the only thing he/she cares about is competing for the opinions of all agents to maximize the consensus and thus maximize his/her benefits. Without the loss of generality, we assume that contestant *A* who holds the opinion of 1 seeks to choose the allocation strategy (*x*_1_, … , *x_n_*) so as to maximize the consensus *C*(*x*_1_, … , *x_n_*, *y*_1_, … , *y_n_*) which is subject to the resource constraint $\sum_{i=1}^{n}x_{i}=R_{A}$. At the same time, contestant *B* who holds the opinion of 0 seeks to choose the allocation strategy (*y*_1_, … , *y_n_*) so as to minimize the consensus *C*(*x*_1_, … , *x_n_*, *y*_1_, … , *y_n_*) which is subject to the resource constraint $\sum_{i=1}^{n}y_{i}=R_{B}$. The social network *G*(*V*, *E*) is fixed and known for both contestants. Following the social exchange theory [53], agents who have obtained resources from the contestant will improve their corresponding opinions as a reward to him/her. Additionally, our model takes initial opinions into consideration while capturing the influence of competitive expenditure on opinions. Taking the election campaign as an example, candidates often try to sway initial opinions about them via a campaign speech. After the speech, people generally update their opinions on candidates through online or offline interaction in social networks. Therefore, we use initial opinions to reflect the asymmetric advantages of contestants in translating their efforts into opinions, as shown in equation (3.5). *x_i_* ∈ (0, *R_A_*) and *y_i_* ∈ (0, *R_B_*) denote the resources allocated to agent *v_i_* by contestants *A* and *B*, respectively.

3.5 $$\begin{array}{rl} \;p_{i}^{0}(0,0) & =p_{i}^{0}\\ \mathrm{and}\;\;\;\;\;\;\;p_{i}^{1}(x_{i},y_{i}) & =\frac{\;p_{i}^{0}x_{i}^{\gamma }}{\;p_{i}^{0}x_{i}^{\gamma }+(1-p_{i}^{0})y_{i}^{\gamma }}, \end{array}\}$$

where 0 < *γ* ≤ 1, $\sum_{i=1}^{n}x_{i}=R_{A}$ and $\sum_{i=1}^{n}y_{i}=R_{B}$.

Based on equation (2.3), the consensus is a weighted linear combination of the consensus weights and the opinions at the beginning of evolution, i.e. $C=\sum_{i=1}^{n}w_{i}p_{i}^{1}$. The consensus weight has been determined by the self-confidence and the network structure (see equation (3.4)). Therefore, in order to compete for agents’ opinions and finally maximize the consensus, contestant *A* or *B* needs to reasonably allocate resources among agents in the process of changing $p_{i}^{0}$ to $p_{i}^{1}$.

Because the function $p_{i}^{1}(x_{i},y_{i})$ is strictly concave in *x_i_* with 0 < *γ* ≤ 1, there exists a unique pure-strategy Nash equilibrium of the competition game that can be regarded as the stable state of opinion evolution in our model [1,54]. From the equilibrium results, the resources allocated to each agent by contestants *A* and *B* in the stable state can be obtained.

**Proposition 3.2.**

*For a given social network G(V, E), contestants A and B make use of their resources, R_A_ and R_B_, to influence opinion evolution over the network. In order to maximize the consensus for them in the stable state, contestants A and B allocate resources to each agent as follows:*

*Contestant A:*

$$x_{i}^{*}=R_{A}\frac{\;p_{i}^{0}(1-p_{i}^{0}){\left(R_{B}/R_{A}\right)}^{\gamma }\left(\sum_{\;j=1,j\neq i}^{n}a_{ij}d_{j}\right)d_{i}/{\left[\;p_{i}^{0}+(1-p_{i}^{0}){\left(R_{B}/R_{A}\right)}^{\gamma }\right]}^{2}(1-\beta_{i})}{\overset{n}{\underset{i=1}{\sum }}\left\{\;p_{i}^{0}(1-p_{i}^{0}){\left(R_{B}/R_{A}\right)}^{\gamma }\left(\sum_{\;j=1,j\neq i}^{n}a_{ij}d_{j}\right)d_{i}/{\left[\;p_{i}^{0}+(1-p_{i}^{0}){\left(R_{B}/R_{A}\right)}^{\gamma }\right]}^{2}(1-\beta_{i})\right\}}.$$

*Contestant B:*

$$y_{i}^{*}=R_{B}\frac{\;p_{i}^{0}(1-p_{i}^{0}){\left(R_{B}/R_{A}\right)}^{\gamma }\left(\sum_{\;j=1,j\neq i}^{n}a_{ij}d_{j}\right)d_{i}/{\left[\;p_{i}^{0}+(1-p_{i}^{0}){\left(R_{B}/R_{A}\right)}^{\gamma }\right]}^{2}(1-\beta_{i})}{\overset{n}{\underset{i=1}{\sum }}\left\{\;p_{i}^{0}(1-p_{i}^{0}){\left(R_{B}/R_{A}\right)}^{\gamma }\left(\sum_{\;j=1,j\neq i}^{n}a_{ij}d_{j}\right)d/{\left[\;p_{i}^{0}+(1-p_{i}^{0}){\left(R_{B}/R_{A}\right)}^{\gamma }\right]}^{2}(1-\beta_{i})\right\}}.$$

*Proof*. The proof of proposition (3.2) is provided in the appendix A.

Proposition (3.2) shows that contestants *A* and *B* allocate the same proportion of resources to each agent in the stable state. However, the amount of resources is different, which is related to their respective total resources. In the stable state, in regard to contestants, the party with more total resources will allocate more resources to the agent. As for agents in the network, given other conditions remain unchanged, an agent with a larger consensus weight will receive more allocated resources.

### 3.3. Consensus under competition in the DGCG model

In the DGCG model, the social network *G*(*V*, *E*) is an undirected graph. According to lemma (3.1), there will be a consensus in a competitive environment in the final stage of opinion evolution.

**Proposition 3.3.**

*Suppose that both contestants A and B compete for opinions of all agents embedded in the social network G(V, E) to maximize their benefits. Each contestant spends its resources on agents to shape their opinions as per equation (3.5). Then, opinions of all agents evolve over the social network through interaction and form a consensus*

3.6 $$C=\frac{\sum_{i=1}^{n}\left\{\;p_{i}^{0}R_{A}^{\gamma }/p_{i}^{0}R_{A}^{\gamma }+(1-p_{i}^{0})R_{B}^{\gamma }\times \left(\sum_{\;j=1,j\neq i}^{n}a_{ij}d_{j}\right)d_{i}/1-\beta_{i}\right\}}{\sum_{i=1}^{n}\left\{\left(\sum_{\;j=1,j\neq i}^{n}a_{ij}d_{j}\right)d_{i}/1-\beta_{i}\right\}}.$$

*Proof.* The proof of proposition (3.3) is provided in the appendix A.

Proposition (3.3) provides the analytic expression of consensus *C*, which shows that the consensus is determined by many factors such as initial opinions, resource ratio of both contestants, network structure and self-confidence. The effect of self-confidence on consensus is relatively simple. Taking self-confidence for example, given that the other conditions are fixed, higher self-confidence of an agent implies that his/her opinion accounts for a larger weight in the consensus. We discuss the effects of the other three factors on the consensus in next section.

## 4. The role of different factors in consensus

This section mainly presents how the resource ratio of both contestants, initial opinions and network structure affect the consensus. The consensus not only shows the final opinion structure under different influencing factors, but also reveals the possible outcome of the competition. To be specific, contestant *A* is the winner if and only if the consensus is greater than 0.5, while contestant *B* is the winner if and only if the consensus is less than 0.5. There is no winner when the consensus is equal to 0.5. Therefore, we define the competition outcome based on the consensus and also discuss the influence of these factors on it.

Based on Propositions (3.1)–(3.3), the effects of these three factors on consensus and competition outcomes are analysed as follows.

### 4.1. Resource ratio of both contestants

In the DGCG model, resources of both contestants can be allocated across agents to change their initial opinions, which is quite different from the SNDG model with no resource expenditure and no competition. Here, we present a theoretical analysis to show what role the resource ratio of both contestants plays in the consensus of the DGCG model. The analysis results are shown in Propositions (4.1) and (4.2).

**Proposition 4.1.**

*In the DGCG model, let ρ denote the resource ratio of contestant A to contestant B, i.e. ρ = R_A_/R_B_. Given the social network G(V, E), the initial opinions of agents and the self-confidence of agents, the consensus C over the social network tends to support contestant A with an increase in the resource ratio ρ. Otherwise, it tends to support contestant B.*

*Proof.* The proof of proposition (4.1) is provided in appendix A.

As proposition (4.1) implies, the resource ratio can influence the consensus over a social network and bias it towards the contestant with greater resources. In addition to the influence on consensus, the resource ratio affects the competition outcome (see proposition (4.2)). Let *S* denote the competition outcome, i.e.

4.1 S=C−(1−C)=2C−1.

Based on the definition of the competition outcome, *S* > 0 means that contestant *A* is the winner, *S* < 0 means that contestant *B* is the winner, and *S* = 0 means that there is no winner in the competition.

**Proposition 4.2.**

*Assume that, initially, all agents share the same opinion p^0^. If R_A_/R_B_ > (1 − p^0^/p^0^)^1/γ^, then contestant A will be the winner in the competition. If 0 < R_A_/R_B_ < (1 − p^0^/p^0^)^1/γ^, then contestant B will be the winner in the competition.*

*Proof.* The proof of proposition (4.2) is provided in the appendix A.

Although proposition (4.2) describes a special situation, it still reveals that the resource ratio of both contestants can influence and even reverse the competition outcome.

### 4.2. Initial opinions

The initial opinion reflects the first impression or inherent cognition of each agent for a contestant. Opinion evolution over the social network is grounded in the initial opinions of agents. We value the role of initial opinions (especially heterogeneous ones) in the allocated resources and consensus at a stable state, which is described in Propositions (4.3) and (4.4).

**Proposition 4.3.**

*For each agent in the social network G(V, E), the resources allocated to agent v_i_ are a concave function of* $p_{i}^{0}$ *that reaches its maximum value at*

$$p_{i}^{0}=\frac{R_{B}^{\gamma }}{R_{B}^{\gamma }+R_{A}^{\gamma }}.$$

*Proof.* The proof of proposition (4.3) is provided in appendix A.

Obviously, Proposition (4.3) indicates that the closer the agent's initial opinion is to $R_{B}^{\gamma }/(R_{B}^{\gamma }+R_{A}^{\gamma })$, the more resources he/she obtains from contestants *A* and *B*. Proposition (4.4) presents some results for the role of initial opinions in the consensus.

**Proposition 4.4.**

*Given that a consensus is reached among the agents according to proposition (3.3), the consensus C increases monotonically on the initial opinion* $p_{i}^{0}$*. Moreover, initial opinions can influence the monotonicity of consensus C on the consensus weight w_i_: (1) if* $p_{i}^{0}=\max\{\;p_{k}^{0}|k\in V\}$*, then the consensus C increases monotonically with the consensus weight w_i_; (2) if* $p_{i}^{0}=\min\{\;p_{k}^{0}|k\in V\}$*, then the consensus C decreases monotonically with the consensus weight w_i_; (3) if* $\min\{\;p_{k}^{0}|k\in V\}<p_{i}^{0}<\max\{\;p_{k}^{0}|k\in V\}$*, then the consensus C is not monotonous with the consensus weight w_i_.*

*Proof.* The proof of proposition (4.4) is provided in the appendix A.

Proposition (4.4) shows that the greater the initial opinion of any agent, the closer the final consensus will be to 1. The initial opinion $p_{i}^{0}$ reveals the agent's first impression of contestants at the beginning of opinion evolution. $0<p_{i}^{0}<0.5$ reflects that agent *v_i_* is an initial supporter of contestant *A*, while $0.5<p_{i}^{0}<1$ reflects that agent *v_i_* is an initial supporter of contestant *B*. In particular, $p_{i}^{0}=0.5$ means that agent *v_i_* neither knows nor supports the two contestants in the beginning. Initial opinions of agents in the social network are often heterogeneous, making it difficult to theoretically analyse the role of initial opinions in the competition outcome. We tend to apply numerical simulations to explore this role.

**Figure 2.**
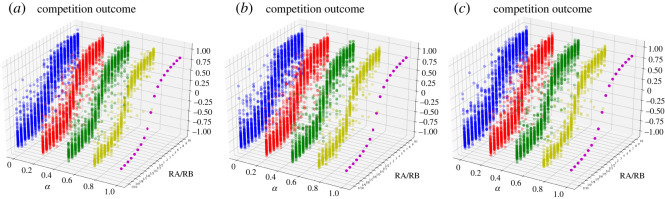
The competition outcomes of 100 realizations under different resource ratios and different values of *α* in (*a*) a random network, i.e. it consists of 20 nodes and the probability for edge creation is 0.5. (*b*) A scale-free network, i.e. it consists of 20 nodes and one edge is created by attaching new nodes to existing nodes. (c) A small-world network, i.e. it consists of 20 nodes, each node is connected with its four nearest neighbours and the probability of rewiring each edge is 0.3.

Given that a social network *G*(*V*, *E*) contains 20 agents, contestants *A* and *B* use their own resources, *R_A_* and *R_B_*, to compete for the preferences of agents in the network. There is a *α* proportion of agents who neither know nor support contestants *A* and *B* in the beginning, i.e. they hold the initial opinion of 0.5. The remaining agents are either the initial supporters of *B* or the initial supporters of *A*, and their initial opinions are uniformly distributed in (0,0.5)∪(0.5,1). The different value of *α* reflects the degree of homophily of agents' initial opinions in the social network. Simulations are carried out to analyse the role of initial opinions in competition outcomes under different resource ratios and different values of *α*. The proportion *α* varies in the scope of [0, 0.25, 0.5, 0.75, 1]. The resource ratio varies in the scope of [1/10,1/9,1/8,1/7,1/6,1/5,1/4,1/3,1/2,1,2,3,4,5,6,7,8,9,10]. Suppose that the parameter *γ* is equal to 1 and self-confidence *β_i_* is generated from pseudorandom numbers uniformly distributed on interval (0, 1). Based on equations (3.2) and (3.5), numerical simulations are realized in the random network [58], the scale-free network [59] and the small-world network [60], respectively. According to equation (4.1), the competition outcome is derived from consensus, which not only represents the results of competition directly but also reflects the level of reached consensus. Thus, we use the competition outcome to represent both of them. The simulation results are shown in figure 2.

From figure 2, the simulation results of three networks indicate that the competition outcomes all show an S-shaped curve with the resource ratio under different values of *α*. Note that the competition outcomes of these three networks are the same, i.e. *S* = *R_A_* − *R_B_*/*R_A_* + *R_B_*, under the value of *α* = 1. On the one hand, the randomness and heterogeneity of initial opinions blur the S-shaped relationship between the competition outcome and the resource ratio. The more homogeneous initial opinions are across all agents, the more ambiguous the S-shaped relationship is. On the other hand, the fewer the agents that hold homogeneous initial opinions, the gentler this S-shaped curve is. This means that the heterogeneity of initial opinions weakens the effectiveness of resources in changing the competition outcome.

Next, the sensitivity analysis of *γ* is conducted in the same small-world network as in figure 2, via simulations.

**Figure 3.**
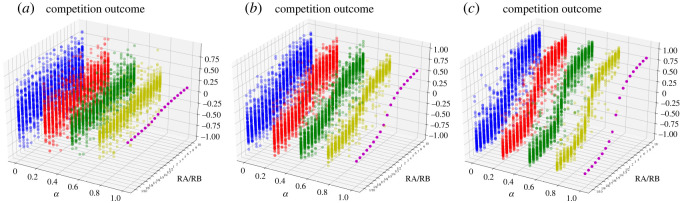
The competition outcomes of 100 realizations under different resource ratios and different values of *α* in the above small-world network with (*a*) *γ* = 0.1, (*b*) *γ* = 0.5 and (*c*) *γ* = 1.

From figure 3, we obtain similar results to figure 2 that the randomness and heterogeneity of initial opinions blur the relationship between the competition outcome and the resource ratio. Furthermore, a small value of *γ* weakens the S-shaped curve between them. According to equations (2.4) and (3.5), *γ* is the efficiency parameter of targeted resources (0 < *γ* ≤ 1). As figure 3 shows, a large value of *γ* means that resources can effectively sway opinions. Conversely, resources are ineffective and will no longer affect the competition outcome when the value of *γ* is infinitely close to 0. This paper highlights the role of resources, and so we set *γ* = 1 for all simulations. For contestants, the value of *γ* is interrelated to the ways and channels of resource consumption. Take marketers for example: the efficiency of marketing resources can be closely related to the mode and content of marketing. This is not our focus, but is worth studying.

**Figure 4.**
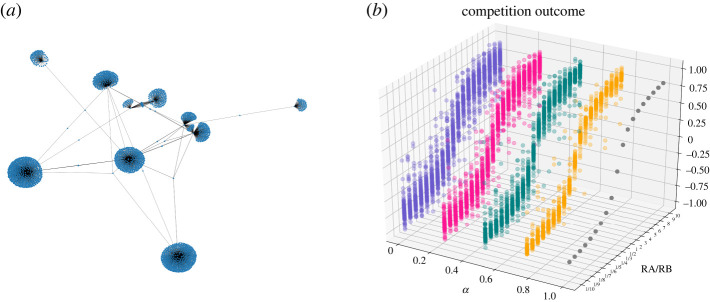
(*a*) The network of Facebook (NIPS). (*b*) The competition outcomes of 100 realizations under different resource ratios and different values of *α* in Facebook (NIPS).

To validate our results, we also consider a real network in the simulations, i.e. Facebook (NIPS). Facebook (NIPS) is an undirected network that contains Facebook user–user friendships. A node represents a Facebook user and an edge indicates that a pair of users are friends on Facebook. The adjacency matrix of Facebook (NIPS) can be downloaded at (http://konect.cc/networks/ego-facebook/), and its basic statistics are presented in table 1. In Facebook (NIPS), there is also an *α* proportion of agents whose initial opinions are 0.5. The initial opinions of other agents are heterogeneous and uniformly distributed in (0,0.5)∪(0.5,1). Meanwhile, other factors and parameters are handled as above. The simulation results of Facebook (NIPS) are shown in figure 4.

**Table 1.** Statistics of Facebook (NIPS). LCC refers to the largest connected component of the network.

network name	no. nodes	no. edges	average degree	maximum degree	size of LCC	clustering coefficient
Facebook (NIPS)	2888	2981	2.064	769	2888	0.000359

The results of figure 4*b* are in line with figure 2, in that the heterogeneity of initial opinions increases the variability of competition outcomes and weakens the effectiveness of targeted resources. More importantly, this shows that our results can be scaled up to real networks.

### 4.3. Network structure

Network structure describes the characteristics of a social network and reflects the relationships of agents embedded in it. What both contestants compete for is the opinion evolving over the social network. Therefore, network structure has a significant influence on consensus and competition outcomes. Although the consensus weight *w_i_* embodies the structure of the social network to some extent (see proposition (3.1)), it cannot give an intuitive expression to the dominance gap of network structure for each contestant. This section highlights the discrepancy of opinion evolution caused by different network structures, which was defined as information gerrymandering in Stewart *et al.* [61]. Stewart *et al*. found that network structure can sway the vote outcome towards one party, even if the status quo of each party is identical [61]. Because they focus on discrete opinions whereas we are concerned with continuous opinions, this paper defines such a situation as the network dominance gap (NDG) to help understand the role of network structure in the consensus and competition outcomes. We define two concepts relating to NDG in both a narrow and broad sense. The detailed definitions are as follows.

**Definition 4.1.**

The social network *G*(*V*, *E*) is an undirected graph consisting of a set *V* of nodes and a set *E* of edges, i.e. *V* = {*v*_1_, *v*_2_, …, *v*_n_}. Let *V*_i_ denote the set of friends of agent *v_i_* *V_i_* = {*j* ∈ *V*|*a_ij_* = 1}. $p_{i}^{0}$ denotes as before. Then, the narrow network dominance of node *v_i_* for contestant *A* is

4.2 $${NG}_{i}^{A}=\underset{\;j\in V_{i}}{\sum }\left(\;p_{j}^{0}\times \frac{d_{j}}{\sum_{k\in V_{i}}d_{k}}\right).$$

The narrow network dominance of node *v_i_* for contestant *B* is

4.3 $${NG}_{i}^{B}=\underset{\;j\in V_{i}}{\sum }\left[(1-p_{j}^{0})\times \frac{d_{j}}{\sum_{k\in V_{i}}d_{k}}\right].$$

Then, the NDG G in the narrow sense is

4.4 $$NG=\overset{n}{\underset{i=1}{\sum }}({NG}_{i}^{A}-{NG}_{i}^{B})=\overset{n}{\underset{i=1}{\sum }}\left\{\frac{\sum_{\;j\in V_{i}}(2p_{j}^{0}-1)d_{j}}{\sum_{\;j\in V_{i}}d_{j}}\right\}.$$

**Definition 4.2.**

The social network *G*(*V*, *E*) is an undirected graph consisting of a set *V* of nodes and a set *E* of edges, i.e. *V* = {*v*_1_, *v*_2_, …, *v*_n_}. Let *V_i_* denote the set of friends of agent *v_i_*, *V_i_* = {*j* ∈ *V*|*a_ij_* = 1}. $p_{i}^{0}$ and *β_i_* denote the same as before. Then, the broad network dominance of node *v_i_* for contestant *A* is

4.5 $$BG_{i}^{A}=(1-\beta_{i})\underset{\;j\in V_{i}}{\sum }\left(\;p_{j}^{0}\times \frac{d_{j}}{\sum_{k\in V_{i}}d_{k}}\right).$$

The broad network dominance of node *v_i_* for contestant *B* is

4.6 $$BG_{i}^{B}=(1-\beta_{i})\underset{\;j\in V_{i}}{\sum }\left[(1-p_{j}^{0})\times \frac{d_{j}}{\sum_{k\in V_{i}}d_{k}}\right].$$

Then, the NDG G in the broad sense is

4.7 $$BG=\overset{n}{\underset{i=1}{\sum }}(1-\beta_{i})(G_{i}^{A}-G_{i}^{B})=\overset{n}{\underset{i=1}{\sum }}\left\{(1-\beta_{i})\frac{\sum_{\;j\in V_{i}}(2p_{j}^{0}-1)d_{j}}{\sum_{\;j\in V_{i}}d_{j}}\right\}.$$

The two concepts of NDG can be regarded as the manifestation of network structure other than the consensus weight. Both of them directly reflect the innate dominance difference of social networks for different contestants and reveal the possible discrepancy of opinion evolution caused by the network structure. We specify that the social network has an innate dominance for contestant *A* at the beginning of opinion evolution if the value of NG (or BG) is positive, and an innate dominance for contestant *B* if the value of NG (or BG) is negative.

More importantly, the narrow NDG is defined by characterizing the intrinsic feature of network structure, and what it captures is the possible discrepancy caused purely by the intrinsic structure. By contrast, a personal attribute, i.e. self-confidence, is accommodated into the broad NDG on the basis of the narrow NDG. What this captures becomes the possible discrepancy jointly caused by the intrinsic structure and the personal attribute. The comparison between these two NDGs highlights the role of self-confidence in the opinion evolution discrepancy caused by network structure, which can provide some inspiration for research into other personal attributes in such a field.

In the following, these two NDGs are employed to discuss the role of network structure in the consensus and competition outcomes. Some theoretical conclusions and simulation results are presented. We first analyse the relationships between these two NDGs and consensus as well as competition outcomes in a simple case where the social network *G*(*V*, *E*) is a regular graph, as shown in proposition (4.5).

**Proposition 4.5.**

*In the regular network G(V, E), let d_0_ denote the degree of each agent. Suppose that both contestants have equal resources, i.e. R_A_ = R_B_ and the self-confidence of each agent is the same, i.e. β_i_ = β_0_. Then, for the narrow NDG, we have* S=(1/n) NG *and* C=(1/2n) NG+1/2 *; for the broad NDG, we have* $S=\frac{BG}{n(1-\beta_{0})}$ *and* $C=\frac{BG}{2n(1-\beta_{0})}+\frac{1}{2}$*.*

*Proof.* The proof of proposition (4.5) is provided in the appendix A.

Although proposition (4.5) describes a simple case, it still provides some insights into the positive relationship between these two NDGs, consensus and competition outcomes. Furthermore, proposition (4.5) indicates that the self-confidence level of network agents can determine the effect of the broad NDG on the consensus and competition outcomes. Specifically, if each agent is highly self-confident, he/she will have less trust in his/her friends. This then leads to a slight opinion evolution in the network, i.e. the resulting opinions may be just slightly different from the initial opinions. Such a highly self-confident agent is also what other researchers refer to as a stubborn agent [38,62].

**Figure 5.**
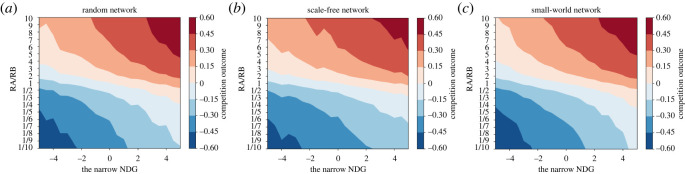
The average competition outcomes of 1000 realizations under different narrow NDGs and different resource ratios in (*a*) a random network, (*b*) a scale-free network and (*c*) a small-world network. (The same networks as figure 2).

**Figure 6.**
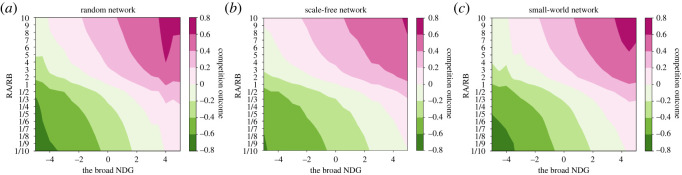
The average competition outcomes of 1000 realizations under different broad NDGs and different resource ratios in (*a*) a random network, (*b*) a scale-free network and (*c*) a small-world network. (The same networks as figure 2).

Next, we simulate a general case where different networks and different resource ratios as well as the heterogeneity of self-confidence are all taken into account. Based on equations (3.6)–(4.2), numerical simulations are carried out in the random network, scale-free network and small-world network, respectively generated as above. All three networks contain 20 agents. The resource ratio varies in the scope of [1/10,1/9,1/8, 1/7, 1/6, 1/5, 1/4, 1/3, 1/2,1,2,3,4,5,6,7,8,9,10]. The self-confidence *β_i_* and the initial opinion $p_{i}^{0}$ of each agent are both generated from pseudorandom numbers uniformly distributed on interval (0, 1). From the simulation results, the relationship between the narrow NDG, the broad NDG and competition outcomes are shown in figures 5 and 6.

From figures 5 and 6, the following common observations are found:

(1) For any given resource ratio, with the increase of these two NDGs from negative to positive, the value of *S* gradually increases. The competition outcome and consensus are both positively correlated with these two NDG. In other words, the more dominant the network structure is for contestant *A* (or *B*), the larger (or smaller) the value of consensus is, and the more likely the competition outcome is that contestant *A* (or *B*) will win.
(2) For any given NDG > 0, *R_A_*/*R_B_* > 1 enhances the innate dominance of network structure over contestant *A* and gradually increases the competition outcome to a positive value, while *R_A_*/*R_B_* < 1 weakens the innate dominance of network structure over contestant *A* and gradually decreases the competition outcome to a negative value. For any given NDG < 0, the opposite is true.

However, there are some differing observations. The positive correlation between the broad NDG and competition outcome is much stronger in figure 6 than between the narrow NDG and competition outcome in figure 5. In other words, the broad NDG has a greater influence on the competition outcome than the narrow NDG. Comparatively, the role of the resource ratio in competition outcomes becomes smaller in figure 6 than in figure 5.

## 5. Conclusion and discussion

The competition for opinions is widely existent in social networks. However, little effort has been devoted to investigating opinion evolution in a competitive environment. In seeking to address this limitation, this paper characterizes a social interaction based on trust relationships and then develops a novel DGCG to explore such a process of opinion evolution as well as consensus-reaching. From the DGCG model, we derive the analytic expression of equilibrium results (final consensus and allocation strategies), which combines the factors of self-confidence, initial opinions and network structure with competitive resources. Similar to [1,37], our model provides a game-theoretic framework for opinion evolution under competition. In addition to that, we comprehensively characterize the black-box of social interaction with the notion of self-confidence, so as to facilitate the analysis of the role of personal attributes and network structure. In brief, our paper makes a valuable contribution to integrating knowledge of psychology and game theory into opinion dynamics.

Furthermore, the role of these factors is analysed in both a theoretical and simulated manner. In line with social exchange theory, we believe that the competitive resources allocated to agents can improve their corresponding opinions [53,63]. Furthermore, we extend it to the case of zero-sum game, where it is the resource ratio of contestants rather than their resource volume that matters in the competition for opinions. We find that the resource ratio can influence the value of consensus, bias it towards the contestant with greater resources, and even reverse the competition outcome. This informs opinion competitors (e.g. candidates in elections and marketers) that the resource gap with opponents dominates the competition, and narrowing the gap can be a way to win. Hence, it is necessary for competitors to investigate and monitor the resources of their opponents in the competition.

In regard to self-confidence and network structure, we find that a highly self-confident agent with many friends, who in turn have many friends, generally holds a large weight in the consensus and obtains a big share of allocated resources. According to [4,23,38], such agents can be regarded as opinion leaders who are undoubtedly the core figures as far as contestants are concerned. This reveals that identification of the core influencers in the network is an important part of contestants’ strategic strategy. We further find that the network structure can have an innate dominance for one of the contestants. Two notions of NDG are created to capture the discrepancy of opinion evolution caused by network structure. The analysis results show that the party with the dominance of network structure is more likely to win the competition for opinions. This provides some useful insights for opinion competitors, i.e. that the network structure can be used as a tool to improve competitive strategy.

In regard to initial opinions, there is an interesting finding. Although each contestant expects agents' initial opinions to be similar to his/hers, he/she does not allocate extensive resources to agents whose initial opinions are very similar or dissimilar to his/hers but to those whose initial opinions are around $R_{B}^{\gamma }/(R_{A}^{\gamma }+R_{B}^{\gamma })$. This suggests that opinion competitors should conduct an initial opinion survey before campaigns and identify the agents with such initial opinions in the network to achieve their strategic objectives. Furthermore, we find that the heterogeneity of initial opinions can weaken the effectiveness of resources in changing the competition outcome and blurs the S-shaped relationship between them. This implies that an initial opinion survey is quite necessary for these competitors.

Despite the above findings, this paper has some limitations. For example, based on the network structure, we consider that an agent puts more trust weight on his/her friends who have more friends. Naturally, our results highlight the influence of network structure on opinion evolution and equilibria. Nevertheless, based on the homophily, other scholars argue that an agent can put more trust weight on his/her friends with more similar opinions [4,64,65]. Therefore, it is possible to explore the influence of individual homophily on opinion evolution. Different from this paper, it will be a nonlinear opinion evolution process. In addition, in our setting, competitive expenditure is applied to change initial opinions so as to indirectly affect opinion dynamics and the resulting opinions of network agents. However, in many practical cases, the influence of resource expenditure on opinions may occur simultaneously with the evolution of opinions [1,66], such as in the case of continuously broadcast product advertising. During advertising, the opinions of individuals are affected not only by the advertisement but also by interaction with other individuals in the network. In other words, resource expenditure can directly affect not only initial opinions, but also opinion dynamics and the resulting opinions. The resource expenditure for each agent becomes time-dependent and state-dependent, which will lead to a hybrid opinion dynamics of linear and nonlinear updating process [39,66]. In addition, this paper formulates a competition for opinions in undirected networks, where agents can accurately express their opinions and become fully aware of the opinions of their friends. However, in practice, social networks are sometimes directed graphs that may cause polarization or fragmentation of opinions. Limited by communication barriers and expressive disorders in reality, agents in the social network cannot become entirely aware of the opinions of others, but only observe the choice of their action. All in all, these limitations indicate avenues for future research to explore.

In future studies, a bounded confidence model can be incorporated into our formulation to capture human homophily [13]. We aim to focus on synchronous opinion evolution combined with dynamic resources allocation in a competitive environment. Such a dynamic game or a repeated game is of great interest. Additionally, the continuous opinions and discrete actions (CODA) model argues that individual inner opinions can be updated according to the observed actions of friends [14]. Thus, competitive opinion dynamics with the CODA model is also a worthy direction for further study.

## Data Availability

See http://doi.org/10.6084/m9.figshare.16912180.

## Appendix A

**A.1. Proof of proposition (3.1)**

*Proof.* The social network *G*(*V*, *E*) is known to be an undirected graph, where any agent can be reached from any other agent via an undirected path. Thus, all agents over the social network will reach a consensus, and the consensus weight *w_i_* of each agent can be obtained by solving the following equations:

$$K\cdot M=K,\;K=(k_{1},\;k_{2},\;\ldots ,\;k_{n}),$$

$$M^{T}\cdot K^{T}=K^{T}$$

$$\mathrm{and}\;\;\;\;\;\;\;\;\;(M^{T}-I)K^{T}=0.$$

Specifically, according to the DGCG model, the above equations can be written as follows:

$$\left\{ \begin{array}{c} (\beta_{1}-1)k_{1}+\frac{(1-\beta_{2})a_{21}d_{1}}{\sum_{\;j=1,j\neq 2}^{n}a_{2j}d_{j}}k_{2}+\cdots +\frac{(1-\beta_{n})a_{n1}d_{1}}{\sum_{\;j=1}^{n-1}a_{nj}d_{j}}k_{n}=0\;\\ \frac{(1-\beta_{1})a_{12}d_{2}}{\sum_{\;j=2}^{n}a_{1j}d_{j}}k_{1}+(\beta_{2}-1)k_{2}+\cdots +\frac{(1-\beta_{n})a_{n2}d_{2}}{\sum_{\;j=1}^{n-1}a_{nj}d_{j}}k_{n}=0\;\\ \frac{(1-\beta_{1})a_{1n}d_{n}}{\sum_{\;j=2}^{n}a_{1j}d_{j}}k_{1}+\frac{(1-\beta_{2})a_{2n}d_{n}}{\sum_{\;j=1,j\neq 2}^{n}a_{2j}d_{j}}k_{2}+\cdots +(\beta_{n}-1)k_{n}=0\; \end{array}\right..$$

There are infinitely many solutions to the above set of equations. However, because the spectral radius of *M^T^* is indeed 1, and following the Perron–Frobenius theorem, the above eigenspace is one-dimensional.

Therefore, all the solutions to the set of equations are in the form *δ* · *k_i_*, where *δ* ∈ *R*, and

$$k_{i}=\frac{\left(\sum_{\;j=1,j\neq i}^{n}a_{ij}d_{j}\right)d_{i}}{1-\beta_{i}}.$$

According to the DeGroot model, we should find a non-negative solution (*w*_1_, … , *w_n_*) such that $\sum_{i=1}^{n}w_{i}=1$. According to the structure of the eigenspace, there is only one such vector; and this vector can be obtained by normalizing the vector *K* = (*k*_1_, *k*_2_, …, *k_n_*). Finally, we have the consensus weight

$$w_{i}=\frac{\left(\sum_{\;j=1,j\neq i}^{n}a_{ij}d_{j}\right)d_{i}/1-\beta_{i}}{\sum_{i=1}^{n}\left\{\left(\sum_{\;j=1,j\neq i}^{n}a_{ij}d_{j}\right)d_{i}/1-\beta_{i}\right\}}.$$

This completes the proof of proposition (3.1).

**A.2. Proof of proposition (3.2)**

*Proof.* In the DGCG model, contestant *A* or *B* wants to maximize the preferences of agents embedded in the social network. In other words, contestant *A* wants to solve

$$\underset{{\left\{x_{i}\right\}}_{i=1}^{n}}{\max}\overset{n}{\underset{i=1}{\sum }}w_{i}\frac{\;p_{i}^{0}x_{i}^{\gamma }}{\;p_{i}^{0}x_{i}^{\gamma }+(1-p_{i}^{0})y_{i}^{\gamma }}$$

and

$$s.t.\;\sum_{i=1}^{n}x_{i}=R_{A}.$$

And contestant *B* wants to solve

$$\underset{{\left\{y_{i}\right\}}_{i=1}^{n}}{\max}\overset{n}{\underset{i=1}{\sum }}w_{i}\frac{(1-p_{i}^{0})y_{i}^{\gamma }}{\;p_{i}^{0}x_{i}^{\gamma }+(1-p_{i}^{0})y_{i}^{\gamma }}$$

and

$$s.t.\;\sum_{i=1}^{n}y_{i}=R_{B}.$$

From the first-order conditions (FOCs), we obtain

For contestant *A*,

$$\frac{\;p_{i}^{0}\gamma x_{i}^{\gamma -1}(1-p_{i}^{0})y_{i}^{\gamma }}{{\left[\;p_{i}^{0}x_{i}^{\gamma }+(1-p_{i}^{0})y_{i}^{\gamma }\right]}^{2}}w_{i}=\lambda_{A};\;\forall i$$

and for contestant *B*,

$$\frac{\;p_{i}^{0}x_{i}^{\gamma }\gamma (1-p_{i}^{0})y_{i}^{\gamma -1}}{{\left[\;p_{i}^{0}x_{i}^{\gamma }+(1-p_{i}^{0})y_{i}^{\gamma }\right]}^{2}}w_{i}=\lambda_{B};\;\forall i$$

From the above two formulae, we have

$$\frac{\lambda_{A}}{\lambda_{B}}=\frac{y_{i}^{*}}{x_{i}^{*}}.$$

Furthermore, because

$$\overset{n}{\underset{i=1}{\sum }}x_{i}=R_{A},\overset{n}{\underset{i=1}{\sum }}y_{i}=R_{B},$$

we can obtain

$$\frac{\lambda_{A}}{\lambda_{B}}=\frac{R_{B}}{R_{A}}.$$

Thus, from the equation

$$\frac{\;p_{i}^{0}\gamma x_{i}^{\gamma -1}(1-p_{i}^{0})y_{i}^{\gamma }}{{\left[\;p_{i}^{0}x_{i}^{\gamma }+(1-p_{i}^{0})y_{i}^{\gamma }\right]}^{2}}w_{i}=\frac{\;p_{i}^{0}\gamma x_{i}^{\gamma -1}(1-p_{i}^{0}){\left((R_{B}R_{A})x_{i}^{*}\right)}^{\gamma }}{{\left[\;p_{i}^{0}x_{i}^{\gamma }+(1-p_{i}^{0}){\left((R_{B}R_{A})x_{i}^{*}\right)}^{\gamma }\right]}^{2}}w_{i}=\lambda_{A},$$

we have:

$$x_{i}^{*}=\frac{\;p_{i}^{0}\gamma (1-p_{i}^{0}){\left(R_{B}R_{A}\right)}^{\gamma }w_{i}}{\lambda_{A}{\left[\;p_{i}^{0}+(1-p_{i}^{0}){\left(R_{B}R_{A}\right)}^{\gamma }\right]}^{2}},\forall i\in V.$$

Similarly, it can be obtained that

$$y_{i}^{*}=\frac{\;p_{i}^{0}\gamma (1-p_{i}^{0}){\left(R_{B}R_{A}\right)}^{\gamma }w_{i}}{\lambda_{B}{\left[\;p_{i}^{0}+(1-p_{i}^{0}){\left(R_{B}R_{A}\right)}^{\gamma }\right]}^{2}},\forall i\in V.$$

Based on

$$R_{A}=\overset{n}{\underset{i=1}{\sum }}x_{i}^{*}=\frac{1}{\lambda_{A}}\overset{n}{\underset{i=1}{\sum }}\left\{\frac{\;p_{i}^{0}\gamma (1-p_{i}^{0}){\left(R_{B}R_{A}\right)}^{\gamma }w_{i}}{{\left[\;p_{i}^{0}+(1-p_{i}^{0}){\left(R_{B}R_{A}\right)}^{\gamma }\right]}^{2}}\right\}\;$$

and

$$R_{B}=\overset{n}{\underset{i=1}{\sum }}y_{i}^{*}=\frac{1}{\lambda_{B}}\overset{n}{\underset{i=1}{\sum }}\left\{\frac{\;p_{i}^{0}\gamma (1-p_{i}^{0}){\left(R_{B}R_{A}\right)}^{\gamma }w_{i}}{{\left[\;p_{i}^{0}+(1-p_{i}^{0}){\left(R_{B}R_{A}\right)}^{\gamma }\right]}^{2}}\right\},$$

we have

$$\lambda_{A}=\frac{1}{R_{A}}\overset{n}{\underset{i=1}{\sum }}\left\{\frac{\;p_{i}^{0}\gamma (1-p_{i}^{0}){\left(R_{B}R_{A}\right)}^{\gamma }w_{i}}{{\left[\;p_{i}^{0}+(1-p_{i}^{0}){\left(R_{B}R_{A}\right)}^{\gamma }\right]}^{2}}\right\},\lambda_{B}=\frac{1}{R_{B}}\overset{n}{\underset{i=1}{\sum }}\left\{\frac{\;p_{i}^{0}\gamma (1-p_{i}^{0}){\left(R_{B}R_{A}\right)}^{\gamma }w_{i}}{{\left[\;p_{i}^{0}+(1-p_{i}^{0}){\left(R_{B}R_{A}\right)}^{\gamma }\right]}^{2}}\right\}.$$

Therefore, we have

$$\begin{array}{rl} & x_{i}^{*}=R_{A}\frac{\;p_{i}^{0}\gamma (1-p_{i}^{0}){\left(R_{B}/R_{A}\right)}^{\gamma }w_{i}/{\left[\;p_{i}^{0}+(1-p_{i}^{0}){\left(R_{B}/R_{A}\right)}^{\gamma }\right]}^{2}}{\overset{n}{\underset{i=1}{\sum }}\left\{\;p_{i}^{0}\gamma (1-p_{i}^{0}){\left(\frac{R_{B}}{R_{A}}\right)}^{\gamma }w_{i}/{\left[\;p_{i}^{0}+(1-p_{i}^{0}){\left(\frac{R_{B}}{R_{A}}\right)}^{\gamma }\right]}^{2}\right\}}\\ & \;y_{i}^{*}=R_{B}\frac{\;p_{i}^{0}\gamma (1-p_{i}^{0}){\left(R_{B}/R_{A}\right)}^{\gamma }wi/{\left[\;p_{i}^{0}+(1-p_{i}^{0}){\left(R_{B}/R_{A}\right)}^{\gamma }\right]}^{2}}{\sum_{i=1}^{n}\{\;p_{i}^{0}\gamma (1-p_{i}^{0}){\left(R_{B}/R_{A}\right)}^{\gamma }w_{i}/{\left[\;p_{i}^{0}+(1-p_{i}^{0}){\left(R_{B}/R_{A}\right)}^{\gamma }\right]}^{2}\}}. \end{array}$$

Furthermore, based on

$$w_{i}=\frac{\left(\sum_{\;j=1,j\neq i}^{n}a_{ij}d_{j}\right)d_{i}/1-\beta_{i}}{\sum_{i=1}^{n}\left\{\left(\sum_{\;j=1,j\neq i}^{n}a_{ij}d_{j}\right)d_{i}/1-\beta_{i}\right\}},$$

we can obtain $x_{i}^{*}$ and $y_{i}^{*}$ shown in proposition (3.2).

This completes the proof of proposition (3.2).

**A.3. Proof of proposition (3.3)**

*Proof.* The proof of proposition (3.3) is based on proposition (3.1) and proposition (3.2). The consensus is $C=\sum_{i=1}^{n}w_{i}p_{i}^{1}$, namely

$$\;C=\overset{n}{\underset{i=1}{\sum }}w_{i}\frac{\;p_{i}^{0}{\left(x_{i}^{*}\right)}^{\gamma }}{\;p_{i}^{0}{\left(x_{i}^{*}\right)}^{\gamma }+(1-p_{i}^{0}){\left(y_{i}^{*}\right)}^{\gamma }}.$$

From proposition (3.1), we have

$$w_{i}=\frac{\left(\sum_{\;j=1,j\neq i}^{n}a_{ij}d_{j}\right)d_{i}/1-\beta_{i}}{\sum_{i=1}^{n}\left\{\left(\sum_{\;j=1,j\neq i}^{n}a_{ij}d_{j}\right)d_{i}/1-\beta_{i}\right\}}.$$

From proposition (3.2), we have

$$\frac{x_{i}^{*}}{y_{i}^{*}}=\frac{R_{A}}{R_{B}}.$$

Then, we can obtain *C* presented in equation (3.6).

This completes the proof of proposition (3.3).

**A.4. Proof of proposition (4.1)**

*Proof.* According to proposition (3.3), we have

$$C=\overset{n}{\underset{i=1}{\sum }}\left\{w_{i}\times \frac{\;p_{i}^{0}R_{A}^{\gamma }}{\;p_{i}^{0}R_{A}^{\gamma }+(1-p_{i}^{0})R_{B}^{\gamma }}\right\},C\in (0,1).$$

From the FOCs, we obtain

$$\frac{\partial C}{\partial \rho }=\overset{n}{\underset{i=1}{\sum }}\left\{\frac{w_{i}p_{i}^{0}(1-p_{i}^{0})\gamma {\left(\rho \right)}^{\gamma -1}}{{\left[\;p_{i}^{0}\rho^{\gamma }+(1-p_{i}^{0})\right]}^{2}}\right\}>0,\forall \rho \in (0,+\infty ).$$

Contestant *A* holds the opinion of 1, and contestant *B* holds the opinion of 0. Therefore, as *ρ* increases, the consensus *C* will be closer to 1; otherwise, it will be closer to 0.

This completes the proof of proposition (4.1).

**A.5. Proof of proposition (4.2)**

*Proof.* For each agent, there is $p_{i}^{0}=p^{0}$. Then, the consensus is

$$C=\overset{n}{\underset{i=1}{\sum }}\left\{w_{i}\times \frac{\;p_{i}^{0}R_{A}^{\gamma }}{\;p_{i}^{0}R_{A}^{\gamma }+(1-p_{i}^{0})R_{B}^{\gamma }}\right\}=\frac{\;p^{0}R_{A}^{\gamma }}{\;p^{0}R_{A}^{\gamma }+(1-p^{0})R_{B}^{\gamma }}\left(\overset{n}{\underset{i=1}{\sum }}w_{i}\right)=\frac{\;p^{0}R_{A}^{\gamma }}{\;p^{0}R_{A}^{\gamma }+(1-p^{0})R_{B}^{\gamma }}.$$

When contestant *A* is the winner in the competition, there exists

$$S=2C-1=\frac{\;p^{0}R_{A}^{\gamma }-(1-p^{0})R_{B}^{\gamma }}{\;p^{0}R_{A}^{\gamma }+(1-p^{0})R_{B}^{\gamma }}>0.$$

Then,

$$\frac{R_{A}}{R_{B}}>{\left(\frac{1-p^{0}}{\;p^{0}}\right)}^{\frac{1}{\gamma }}.$$

Similarly, when contestant *B* is the winner, there exists

S=2C−1<0.

Then,

$$\frac{R_{A}}{R_{B}}<{\left(\frac{1-p^{0}}{\;p^{0}}\right)}^{\frac{1}{\gamma }}.$$

This completes the proof of proposition (4.2).

**A.6. Proof of proposition (4.3)**

*Proof.* According to proposition (3.2), we have

$$\frac{\partial x_{i}^{*}}{\partial p_{i}^{0}}=R_{A}\frac{{\left(R_{B}/R_{A}\right)}^{\gamma }w_{i}[(1-p_{i}^{0}){\left(R_{B}/R_{A}\right)}^{\gamma }-p_{i}^{0}]/{\left[\;p_{i}^{0}+(1-p_{i}^{0}){\left(R_{B}/R_{A}\right)}^{\gamma }\right]}^{2}\times \overset{n}{\underset{\;j=1,j\neq i}{\sum }}\{\;p_{j}^{0}(1-p_{j}^{0}){\left(R_{B}/R_{A}\right)}^{\gamma }w_{j}/{\left[\;p_{j}^{0}+(1-p_{j}^{0}){\left(R_{B}/R_{A}\right)}^{\gamma }\right]}^{2}\}}{{\left\{\overset{n}{\underset{i=1}{\sum }}p_{i}^{0}(1-p_{i}^{0}){\left(R_{B}/R_{A}\right)}^{\gamma }w_{i}/{\left[\;p_{i}^{0}+(1-p_{i}^{0}){\left(R_{B}/R_{A}\right)}^{\gamma }\right]}^{2}\right\}}^{2}},$$

and

$$\frac{\partial y_{i}^{*}}{\partial p_{i}^{0}}=R_{B}\frac{{\left(R_{B}/R_{A}\right)}^{\gamma }w_{i}[(1-p_{i}^{0}){\left(R_{B}/R_{A}\right)}^{\gamma }-p_{i}^{0}]/{\left[\;p_{i}^{0}+(1-p_{i}^{0}){\left(R_{B}/R_{A}\right)}^{\gamma }\right]}^{2}\times \overset{n}{\underset{\;j=1,j\neq i}{\sum }}\{\;p_{j}^{0}(1-p_{j}^{0}){\left(R_{B}/R_{A}\right)}^{\gamma }w_{j}/{\left[\;p_{j}^{0}+(1-p_{j}^{0}){\left(R_{B}/R_{A}\right)}^{\gamma }\right]}^{2}\}}{{\left\{\overset{n}{\underset{i=1}{\sum }}p_{i}^{0}(1-p_{i}^{0}){\left(R_{B}/R_{A}\right)}^{\gamma }w_{i}/{\left[\;p_{i}^{0}+(1-p_{i}^{0}){\left(R_{B}/R_{A}\right)}^{\gamma }\right]}^{2}\right\}}^{2}},$$

When $p_{i}^{0}\in (0,\;(\;R_{B}^{\gamma }/R_{B}^{\gamma }+R_{A}^{\gamma })\;\;)$, then $\partial x_{i}^{*}/\partial p_{i}^{0}>0$ and $\partial y_{i}^{*}/\partial p_{i}^{0}>0$. When $p_{i}^{0}\in (R_{B}^{\gamma }/(R_{B}^{\gamma }+R_{A}^{\gamma }),\;1)$, then $\partial x_{i}^{*}/\partial p_{i}^{0}<0$ and $\partial y_{i}^{*}/\partial p_{i}^{0}<0$. Therefore, the resources that agent *v_i_* obtains from contestants *A* and *B* reach the maximum value, respectively, while $p_{i}^{0}$ is equal to $R_{B}^{\gamma }/(R_{B}^{\gamma }+R_{A}^{\gamma })$.

This completes the proof of proposition (4.2).

**A.7. Proof of proposition (4.3)**

*Proof.* From the FOCs for equation (3.6), we have

$$\frac{\partial C}{\partial p_{i}^{0}}=\frac{R_{A}^{\gamma }R_{B}^{\gamma }w_{i}}{{\left[\;p_{i}^{0}R_{A}^{\gamma }+(1-p_{i}^{0})R_{B}^{\gamma }\right]}^{2}}>0.$$

Thus, consensus increases monotonically on the initial opinion.

Owing to $\sum_{i=1}^{n}w_{i}=1$, the consensus opinion *C* can be written as

$$C=\overset{n}{\underset{i=1,i\neq j}{\sum }}\left(\frac{\;p_{i}^{0}R_{A}^{\gamma }}{\;p_{i}^{0}R_{A}^{\gamma }+(1-p_{i}^{0})R_{B}^{\gamma }}w_{i}\right)+\frac{\;p_{j}^{0}R_{A}^{\gamma }}{\;p_{j}^{0}R_{A}^{\gamma }+(1-p_{j}^{0})R_{B}^{\gamma }}\left(1-\overset{n}{\underset{i=1,i\neq j}{\sum }}w_{i}\right).$$

From the FOCs for *A*, we obtain

$$\frac{\partial C}{\partial w_{i}}=\frac{\;p_{i}^{0}R_{A}^{\gamma }}{\;p_{i}^{0}R_{A}^{\gamma }+(1-p_{i}^{0})R_{B}^{\gamma }}-\frac{\;p_{j}^{0}R_{A}^{\gamma }}{\;p_{j}^{0}R_{A}^{\gamma }+(1-p_{j}^{0})R_{B}^{\gamma }},(i\neq j).$$

For the function

$$h(\alpha )=\frac{\alpha R_{A}^{\gamma }}{\alpha R_{A}^{\gamma }+(1-\alpha )R_{B}^{\gamma }},(\gamma \in (0,1],R_{A}\in R^{+},R_{B}\in R^{+}),$$

there is

$$\frac{\partial h}{\partial \alpha }=\frac{R_{A}^{\gamma }R_{B}^{\gamma }}{{\left[\alpha R_{A}^{\gamma }+(1-\alpha )R_{B}^{\gamma }\right]}^{2}}>0.$$

Then, the function *h*(*α*) is monotonically increasing with *α* ∈ (0, 1).

Therefore, if $p_{i}^{0}=\max\{\;p_{k}^{0}|k\in V\}$, then

$$\frac{\partial C}{\partial w_{i}}=\frac{\;p_{i}^{0}R_{A}^{\gamma }}{\;p_{i}^{0}R_{A}^{\gamma }+(1-p_{i}^{0})R_{B}^{\gamma }}-\frac{\;p_{j}^{0}R_{A}^{\gamma }}{\;p_{j}^{0}R_{A}^{\gamma }+(1-p_{j}^{0})R_{B}^{\gamma }}>0.$$

If $p_{i}^{0}=\min\{\;p_{k}^{0}|k\in V\}$, then

$$\frac{\partial C}{\partial w_{i}}=\frac{\;p_{i}^{0}R_{A}^{\gamma }}{\;p_{i}^{0}R_{A}^{\gamma }+(1-p_{i}^{0})R_{B}^{\gamma }}-\frac{\;p_{j}^{0}R_{A}^{\gamma }}{\;p_{j}^{0}R_{A}^{\gamma }+(1-p_{j}^{0})R_{B}^{\gamma }}<0.$$

However, when $\min\{\;p_{k}^{0}|k\in V\}<p_{i}^{0}<\max\{\;p_{k}^{0}|k\in V\}$, we cannot judge the relationship between ∂*C*/∂*w_i_* and 0.

This completes the proof of proposition (4.3).

**A.8. Proof of proposition (4.4)**

*Proof.* The above conditions are known, then the consensus *C* can be simplified as

$$C=\frac{d_{0}^{3}/1-\beta_{0}\times \sum_{i=1}^{n}p_{i}^{0}}{nd_{0}^{3}/1-\beta_{0}}=\frac{1}{n}\overset{n}{\underset{i=1}{\sum }}p_{i}^{0}.$$

Thus, the competition outcome *S* is

$$S=\frac{1}{n}\overset{n}{\underset{i=1}{\sum }}(2p_{i}^{0}-1).$$

Meanwhile, the narrow NDG NG is

$$NG=\overset{n}{\underset{i=1}{\sum }}\left\{\frac{\sum_{\;j\in V_{i}}(2p_{j}^{0}-1)d_{0}}{d_{0}^{2}}\right\}=\overset{n}{\underset{i=1}{\sum }}(2p_{i}^{0}-1).$$

and the broad NDG BG is

$$BG=\overset{n}{\underset{i=1}{\sum }}\left\{(1-\beta_{0})\frac{\sum_{\;j\in V_{i}}(2p_{j}^{0}-1)d_{0}}{d_{0}^{2}}\right\}=(1-\beta_{0})\overset{n}{\underset{i=1}{\sum }}(2p_{i}^{0}-1).$$

Therefore, for the narrow NDG, S=(1/n)NG and C=(1/2n NG+1/2.

For the broad NDG, $S=BG/n(1-\beta_{0})$ and $C=BG/2n(1-\beta_{0})+1/2$.

This completes the proof of proposition (4.4).
